# Impact of age at type 2 diabetes mellitus diagnosis on mortality and vascular complications: systematic review and meta-analyses

**DOI:** 10.1007/s00125-020-05319-w

**Published:** 2020-12-14

**Authors:** Natalie Nanayakkara, Andrea J. Curtis, Stephane Heritier, Adelle M. Gadowski, Meda E. Pavkov, Timothy Kenealy, David R. Owens, Rebecca L. Thomas, Soon Song, Jencia Wong, Juliana C.-N. Chan, Andrea O.-Y. Luk, Giuseppe Penno, Linong Ji, Viswanathan Mohan, Anandakumar Amutha, Pedro Romero-Aroca, Danijela Gasevic, Dianna J. Magliano, Helena J. Teede, John Chalmers, Sophia Zoungas

**Affiliations:** 1 School Public Health and Preventive Medicine, Monash University, Melbourne, VIC Australia; 2 Baker Heart and Diabetes Institute, Melbourne, VIC Australia; 3 Centers for Disease Control and Prevention, Division for Diabetes Translation, Atlanta, GA USA; 4 Department of Medicine, University of Auckland, Auckland, New Zealand; 5 Diabetes Research Group, Swansea University Medical School, Swansea, Wales UK; 6 Department of Diabetes, Northern General Hospital, Sheffield, UK; 7 Royal Prince Alfred Hospital, Camperdown, NSW Australia; 8 Department of Medicine and Therapeutics, The Chinese University of Hong Kong, Hong Kong Special Administrative Region, Shenzhen, People’s Republic of China; 9 Diabetes and Metabolic Disease Section, Department of Clinical and Experimental Medicine, Azienda Ospedaliero-Universitaria Pisana University of Pisa, Pisa, Italy; 10 Department of Endocrinology, Peking University People’s Hospital, Xicheng District, Beijing, China; 11 Madras Diabetes Research Foundation & Dr Mohan’s Diabetes Specialities Centre, Chennai, India; 12 Hospital Universtario Sant Joan, Reus, Spain; 13 Usher Institute, University of Edinburgh, Old Medical School, Teviot Place, Edinburgh, UK; 14 Monash Centre for Health Research and Implementation, Monash University, Clayton, VIC Australia; 15 The George Institute for Global Health, Camperdown, NSW Australia

**Keywords:** Age factors, Age of onset, Diabetes, Diabetes complications, Diabetes mellitus, type 2, Disease progression, Meta-analysis, Prognosis, Systematic review

## Abstract

**Aims/hypothesis:**

Few studies examine the association between age at diagnosis and subsequent complications from type 2 diabetes. This paper aims to summarise the risk of mortality, macrovascular complications and microvascular complications associated with age at diagnosis of type 2 diabetes.

**Methods:**

Data were sourced from MEDLINE and All EBM (Evidence Based Medicine) databases from inception to July 2018. Observational studies, investigating the effect of age at diabetes diagnosis on macrovascular and microvascular diabetes complications in adults with type 2 diabetes were selected according to pre-specified criteria. Two investigators independently extracted data and evaluated all studies. If data were not reported in a comparable format, data were obtained from authors, presented as minimally adjusted ORs (and 95% CIs) per 1 year increase in age at diabetes diagnosis, adjusted for current age for each outcome of interest. The study protocol was recorded with PROSPERO International Prospective Register of Systematic Reviews (CRD42016043593).

**Results:**

Data from 26 observational studies comprising 1,325,493 individuals from 30 countries were included. Random-effects meta-analyses with inverse variance weighting were used to obtain the pooled ORs. Age at diabetes diagnosis was inversely associated with risk of all-cause mortality and macrovascular and microvascular disease (all *p* < 0.001). Each 1 year increase in age at diabetes diagnosis was associated with a 4%, 3% and 5% decreased risk of all-cause mortality, macrovascular disease and microvascular disease, respectively, adjusted for current age. The effects were consistent for the individual components of the composite outcomes (all *p* < 0.001).

**Conclusions/interpretation:**

Younger, rather than older, age at diabetes diagnosis was associated with higher risk of mortality and vascular disease. Early and sustained interventions to delay type 2 diabetes onset and improve blood glucose levels and cardiovascular risk profiles of those already diagnosed are essential to reduce morbidity and mortality.

**Figure:**
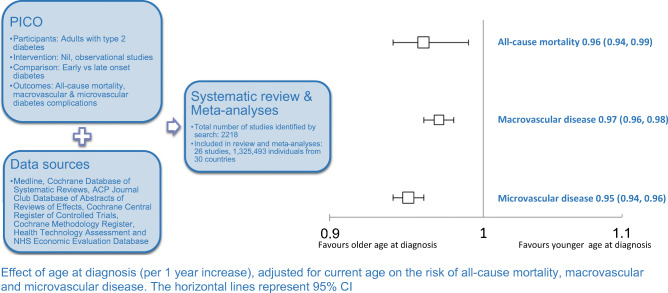
Graphical abstract

**Supplementary Information:**

The online version contains supplementary material available at 10.1007/s00125-020-05319-w.

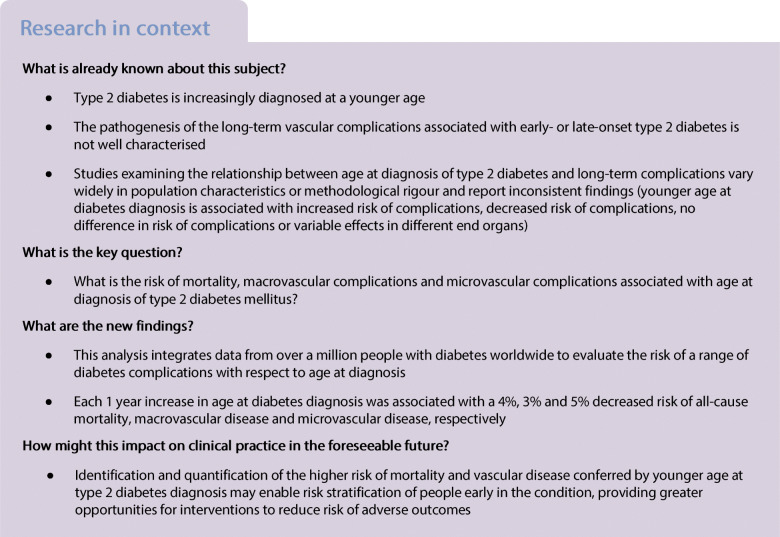



## Introduction

The IDF estimates that the prevalence of diabetes will rise from 425 million people worldwide in 2017, to 629 million by 2045 [1]. Type 2 diabetes, conventionally considered a disease of middle and older age, is increasingly diagnosed at a younger age [1, 2]. Type 2 diabetes and its associated complications contribute to 8.4% of deaths worldwide, consuming significant healthcare resources [3]; this is likely to rise exponentially given the increasing prevalence of the condition [1].

Despite significant diagnostic, monitoring and treatment advances, type 2 diabetes remains associated with increased mortality and morbidity compared with the general population [4]. However, the pathogenesis of the long-term vascular complications associated with early- or late-onset type 2 diabetes is not well characterised and although the mechanisms for the development of complications may be similar [5], recent evidence suggests an accelerated course in people diagnosed with early-onset type 2 diabetes [6, 7]. Proposed mechanisms include a longer lifetime exposure to the adverse diabetic milieu and/or early-onset type 2 diabetes representing an inherently more aggressive metabolic phenotype with rapid onset of beta cell failure and insulin resistance compared with late-onset disease [2, 8, 9]. Novel cluster analyses raise the possibility of type 2 diabetes representing a clustering of up to five disease subgroups with distinct age at diagnosis, genetics, mechanisms of disease progression and risk of diabetic complications [10]. Of the five groups identified, the ‘mild age-related diabetes’ subgroup contains elderly people who experience the most benign disease course compared with the ‘mild obesity-related diabetes’ group, characterised by younger age at onset and obesity.

Several studies have examined the relationship between age at diabetes diagnosis and long-term complications among people with type 2 diabetes. These studies vary widely in population characteristics and methodological rigour and report inconsistent findings (e.g. younger age at diabetes diagnosis is associated with increased risk of complications [6, 7, 11–15], decreased risk of complications [16, 17], no difference in risk of complications [18] or variable effects in different end organs [19, 20]). Additionally, some studies have proposed that longer diabetes duration [21, 22] or more adverse cardiovascular risk profiles [23, 24] underlie the greater risk of development of vascular complications associated with type 2 diabetes diagnosed at a younger age, while other studies have suggested that impact of age at diagnosis may vary with ethnicity [25].

Evidence of a clinically meaningful effect of age at diagnosis of type 2 diabetes beyond the ageing process itself would have substantial implications for diabetes prevention and treatment as well as the development and implementation of cardiovascular risk prediction tools. The aim of our study was thus to examine the effect of age at diagnosis of type 2 diabetes on risks of complications, focusing on all-cause mortality, macrovascular events and microvascular events.

## Methods

### Data sources and searches

A systematic search of published literature was conducted in MEDLINE and All EBM (Evidence Based Medicine; www.ovid.com/product-details.904.html) databases (including Cochrane Database of Systematic Reviews, ACP Journal Club Database of Abstracts of Reviews of Effects, Cochrane Central Register of Controlled Trials, Cochrane Methodology Register, Health Technology Assessment and NHS Economic Evaluation Database) using the subject headings and key terms detailed in electronic supplementary materials (ESM) Methods. The study methods and reporting follow the Meta-analyses Of Observational Studies in Epidemiology (MOOSE) and Preferred Reporting Items for Systematic review and Meta-Analysis (PRISMA) guidelines [26, 27]. The study protocol was recorded with PROSPERO international prospective register of systematic reviews (CRD42016043593). The search was limited to humans and English language articles and was initially conducted in July 2016 with no time restrictions and updated in July 2018.

### Study selection

The inclusion criteria were determined a priori (ESM Table 1). To be included, studies had to meet the following criteria: be a study of adult participants with type 2 diabetes investigating the effect of age at diabetes diagnosis on macrovascular and microvascular diabetes complications; the study had to assess one or more of the outcome variables all-cause mortality, macrovascular disease, microvascular disease, retinopathy, nephropathy, neuropathy, CVD, cerebrovascular disease and peripheral vascular disease; the study had to have an available mortality/complication rate, where mortality was either a pre-specified primary or secondary outcome, or the methods indicated complete follow-up of participants.

Two independent authors (NN and AMG) assessed the title and abstracts of retrieved records for relevance and duplication. Authors then reviewed the full text of potentially eligible citations to identify studies that fulfilled the inclusion criteria. Any uncertainties regarding study inclusion and data extraction were discussed with an experienced systematic reviewer (AJC), statistician (SH) and senior clinician (SZ). The references cited in the retrieved publications were screened for potentially eligible studies. When several articles from the same study had reported on the same endpoint, only the data representing the longest follow-up were extracted.

### Data extraction and quality assessment

Data were extracted from included studies using a specially developed data extraction form. Information was obtained regarding study design and location, participant characteristics, outcome variables and results. Given the wide variation in data reporting and adjustment for confounders, meaningful interpretation, comparison and meta-analysis was not possible. Therefore, we contacted authors to re-analyse and present data in a homogeneous format to enable data pooling and comparison. Corresponding authors were contacted by e-mail at least twice (if data were not reported in a suitable format) to request data, presented as minimally adjusted ORs (95% CI) per 1 year increase in age at diabetes diagnosis, with adjustment for current age (or diabetes duration) for each outcome of interest. This format was chosen as most of the studies presented the results in this way.

Risk of bias of included studies was assessed using a specially developed data extraction form, based on the Newcastle–Ottawa scaling for non-randomised studies [28, 29]. Quality assessment criteria included representativeness of participants, validity of the diagnostic criteria, determination of age at diagnosis, outcome assessment, withdrawals and losses to follow-up. Each study was then allocated a risk of bias rating (ESM Tables 2, 3).

### Exposures

Current age was reported by each study as age at entry into the study or age at baseline assessment. Age at type 2 diabetes diagnosis was reported as the age of the people at the diagnosis of type 2 diabetes, with diabetes duration reported or calculated as current age minus age at diabetes diagnosis.

### Outcomes

The primary a priori outcomes were all-cause mortality, macrovascular disease (composite of CHD, cerebrovascular disease and peripheral vascular disease) or microvascular disease (composite of retinopathy, nephropathy and neuropathy). The secondary a priori outcomes were retinopathy, nephropathy, neuropathy, CHD, cerebrovascular disease and peripheral vascular disease.

### Statistical analysis

Interdependence of current age, age at diabetes diagnosis and diabetes duration precluded use of all three variables in the same model; hence the use of models containing either age at diabetes diagnosis adjusted for current age or age at diabetes diagnosis adjusted for diabetes duration. Adjustment for current age was to remove the effect of ageing per se. Adjustment for diabetes duration was to remove the effect of the time point at which observations happened to be made in the course of illness for each individual; an individual observed early in their illness would appear to have a longer time to develop complications than the same individual observed late in their illness. For studies reporting in multiple models, we extracted data for both minimally adjusted and maximally adjusted increased risk estimates. Unless otherwise stated, the least adjusted risk estimates from each study were used, provided diabetes duration was included. Review Manager (RevMan) software Version 5.3 was used for all statistical analyses [30].

Data were combined in meta-analyses to calculate pooled risk estimates presented as OR (95% CI) of the effect of age at diabetes diagnosis (per year) adjusted for current age (or diabetes duration [supplemental analyses]), on outcomes using both fixed and random-effects models (generic inverse variance method) [31]. There were no significant differences between fixed- and random-effects analyses. Random-effects models are presented given heterogeneity among the studies [32]. Crude data were included where possible, given variable control for confounding factors. However, some articles presented adjusted ORs only.

*I*^2^ was used to assess heterogeneity with values of 25%, 50% and 75% considered low, moderate and high, respectively [33]. Funnel plots were used to explore potential publication bias [34, 35]. A scatter plot of the *t* statistic associated with each study-estimate value assessed the contribution of each study to the study-estimate random effect vs the log_10_ of the SE of the effect.

## Results

### Characteristics of included studies

**Fig. 1:**
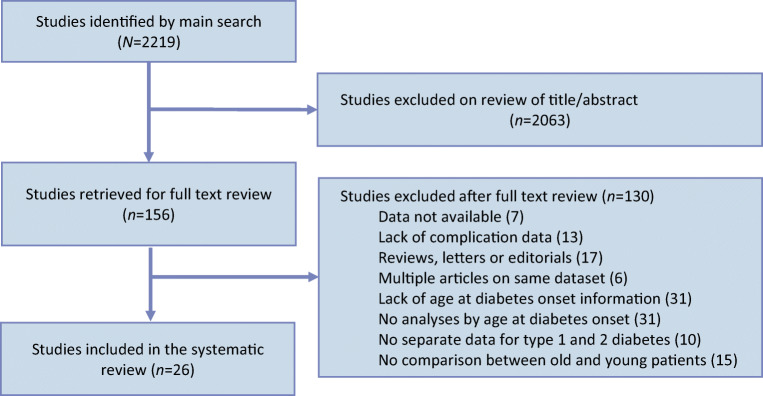
Flow diagram of study selection process

Electronic database and reference searching yielded 2219 publications, of which 156 were reviewed in full text (Fig. 1). Of 33 eligible studies, 26 studies comprising 1,325,493 individuals were included and seven were excluded because data were not provided in the required format in the publication and attempts to contact authors were not successful. The 26 included studies were either cross-sectional (13 studies) or cohort (13 studies) in design. The updated search in 2018 enabled the inclusion of data from three studies. The mean age of study participants ranged from 21.6 years to 67.4 years. The proportion of female study participants ranged from 42.5% to 68.6%. Table 1 summarises the characteristics of the included studies, which comprise 1,325,493 participants from 30 countries worldwide.

**Table 1:** Characteristics of included studies

Study	Year published	Study period	Country	Sample size^a^	Mean age (years)	Sex (% female)	Study design	Outcome
Amutha et al [41]	2017	2001–ongoing	India	198	21.6	42.7	Cohort	Retinopathy, neuropathy, nephropathy, peripheral vascular disease
Amutha et al [43]	2011	1992–2009	India	2630	28.2	NR	Cohort	Retinopathy, neuropathy, nephropathy
Cai et al [14]	2014	2004–2011	China	3100	57.1	60.7	Cohort	Retinopathy
Chan et al [38]	2014	1995–2009	China	9506	57.4	53.8	Cohort	Macrovascular disease, microvascular disease, CHD, cerebrovascular disease, peripheral vascular disease, retinopathy, nephropathy, neuropathy
Chen et al [53]	1992	1985–1986	Taiwan	527	NR	55.0	Cross-sectional	Retinopathy
Hamman et al [54]	1989	1984–1986	USA	251	NR	NR	Cross-sectional	Retinopathy
Huo et al [52]	2018	1997–2011	Australia	743,709	60.2	46.0	Cohort	All-cause mortality
Huo et al [37]	2016	2012	China	222,770	58.3	46.0	Cross-sectional	Microvascular disease, macrovascular disease, retinopathy, nephropathy
Kenealy et al [25]	2008	2000–2005	New Zealand	67,563	60.5	51.0	Cohort	Macrovascular disease, CHD, cerebrovascular disease, peripheral vascular disease
Nanayakkara et al [39]	2018	2015	Australia	3419	62.9	46.1	Cross-sectional	Macrovascular disease, microvascular disease, CHD, cerebrovascular disease, peripheral vascular disease, retinopathy, nephropathy, peripheral neuropathy
Pavkov et al [21]	2006	1965–2002	USA	3653	40.9	61.9	Cohort	All-cause mortality, nephropathy
Pradeepa et al [45]	2008	2001–ongoing	India	1629	50.4	55.4	Cross-sectional	Neuropathy
Pradeepa et al [44]	2014	2001–ongoing	India	1755	50.7	56.2	Cross-sectional	Peripheral vascular disease
Pradeepa et al [42]	2010	2001–ongoing	India	1608	NR	NR	Cross-sectional	Microvascular disease
Penno et al [36]	2018	2006–2008	Italy	15,773	66.6	43.1	Longitudinal	All-cause mortality
Pugliese et al [40]	2012	2007–2008	Italy	15,933	66.2	43.7	Cross-sectional	Macrovascular disease, CHD, cerebrovascular disease, retinopathy
Rema et al [46]	2005	2001–ongoing	India	1715	52.0	55	Cross-sectional	Retinopathy
Romero-Aroca et al [47]	2017	2007–2017	Spain	15,030	65.6	43.8	Longitudinal	Retinopathy, nephropathy
Song and Hardisty [23]	2009	2008	UK	2733	64.2	NR	Cross-sectional	Macrovascular disease, CHD, cerebrovascular disease, peripheral vascular disease, neuropathy, retinopathy
Song and Gray [48]	2011	NR	UK	2516	63.1	NR	Cross-sectional	Retinopathy
Thomas et al [49]	2015	2005–2009	UK	152,156^b^	67.4	68.6	Cohort	Retinopathy
Unnikrishnan et al [50]	2017	2005–2009	India	534	31.6 and 69.9	NR	Cohort	Retinopathy, neuropathy, nephropathy, peripheral vascular disease
Unnikrishnan et al [51]	2007	2005–2009	India	1716	50.7	55.2	Cross-sectional	Nephropathy
Wong et al [15]	2008	1989–2007	Australia	1476	65.0^c^	44.6	Cohort	Retinopathy
Yeung et al [24]	2014	2007–2012	Multinational^d^	42,453	57.5	47.0	Cross-sectional	Retinopathy, neuropathy, nephropathy
Zoungas et al [20]	2014	2001–2008	Multinational^e^	11,140	65.8	42.5	Cohort	All-cause mortality, macrovascular disease, microvascular disease, CHD, cerebrovascular disease, retinopathy, nephropathy

^a^Numbers may vary slightly per outcome analysed, refer to the relevant meta-analysis

^b^The original publication provided results from 2005–2009 (*n* = 86,390); ORs in this meta-analysis are from updated data (unpublished: provided by Rebecca L. Thomas and David R. Owens) with results from 2005–2013 (*n* = 152,156)

^c^Age at last examination

^d^Hong Kong, China, India, Philippines, South Korea, Vietnam, Singapore, Thailand, Taiwan

^e^Australia, Canada, China, Czech, Republic, Estonia, France, Germany, Hungary, India, Ireland, Italy, Lithuania, Malaysia, Netherlands, New Zealand, Philippines, Poland, Russia, Slovakia, UK

NR, Not reported

### Primary outcomes

#### Effects of age at diabetes diagnosis adjusted for current age on all-cause mortality, macrovascular disease and microvascular disease

**Fig. 2:**
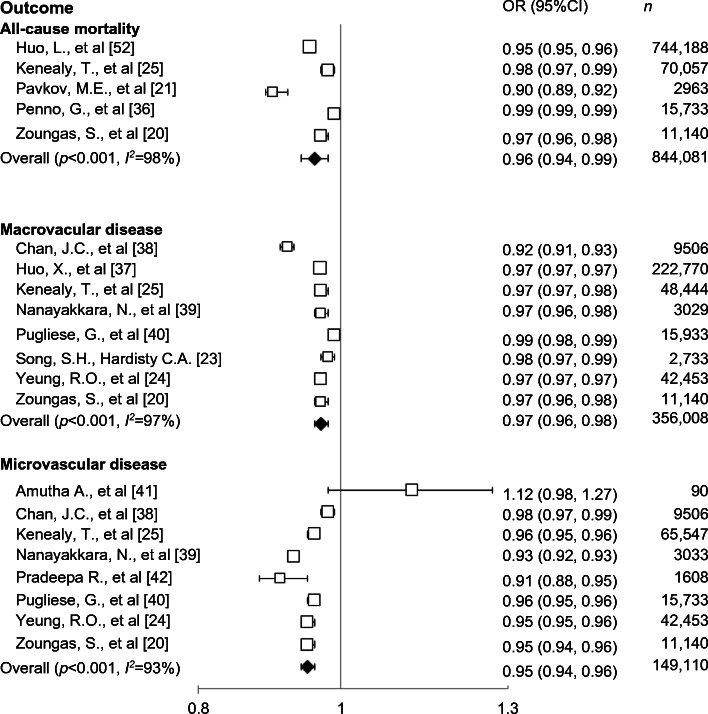
Effect of age at diagnosis (per 1 year increase), adjusted for current age on the risk of all-cause mortality, macrovascular disease and microvascular disease. The size of the symbols is proportional to the study weight and horizontal lines represent 95% CIs

For all-cause mortality, data from five studies [20, 21, 25, 36, 37], comprising 1,325,493 participants, indicated that each 1 year increase in age at diabetes diagnosis was associated with a 4% decreased risk of all-cause mortality (OR 0.96 [0.94, 0.99], *p* < 0.001) when adjusted for current age. For macrovascular disease, data from eight studies [20, 23–25, 37–40], comprising 566 011 participants, indicated that each 1 year increase in age at diabetes diagnosis was associated with a 3% decreased risk of macrovascular disease (OR 0.97 [0.96, 0.98], *p* < 0.001) when adjusted for current age. For microvascular disease, data from eight studies [20, 24, 25, 38–42], comprising 149,110 participants, indicated that each 1 year increase in age at diabetes diagnosis was associated with a 5% decreased risk of microvascular disease (OR 0.95 [0.94, 0.96], *p* < 0.001) when adjusted for current age. Significant heterogeneity in the magnitude of the effects was evident between studies for these outcomes (all *χ*^2^ *p* < 0.001, all *I*^2^ ≥ 93%) (Fig. 2).

### Secondary outcomes

#### Effects of age at diabetes diagnosis adjusted for current age on CHD, cerebrovascular disease, peripheral vascular disease, retinopathy, nephropathy and neuropathy

**Fig. 3:**
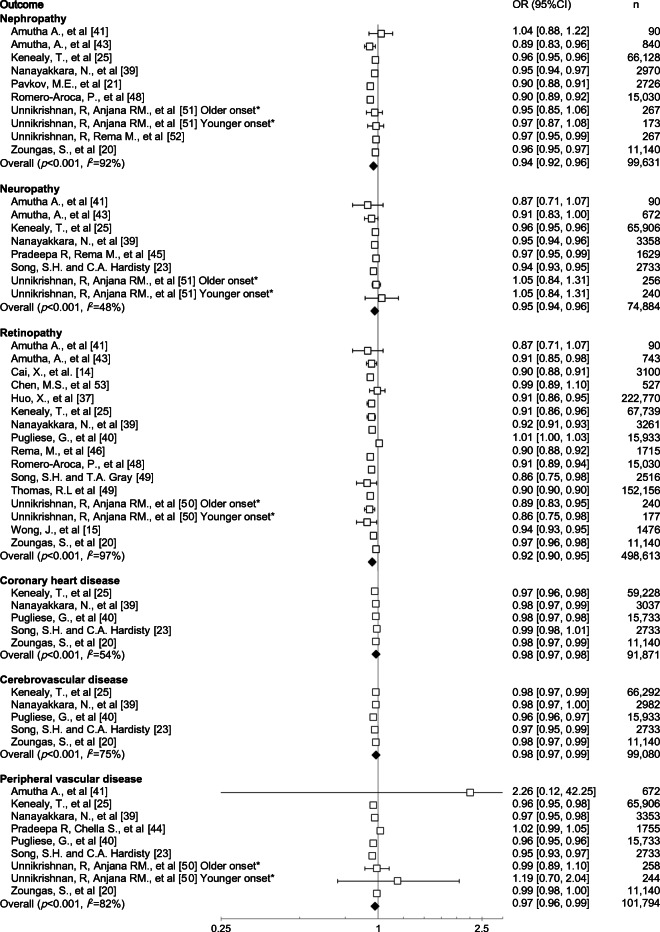
Effect of age at diagnosis (per 1 year increase), adjusted for current age on the risk of secondary outcomes. The symbols are proportional to the study weight and horizontal lines represent 95% CIs. For Unnikrishnan, R, Anjana RM., et al [50], older onset refers to those diagnosed aged >50 years and younger onset refers to those diagnosed aged ≤25 years

Data for individual vascular complications were available from 13 studies, comprising 566 011 participants and adjusted for current age. Each 1 year increase in age at diabetes diagnosis was associated with a 2% decreased risk of CHD (OR 0.98 [95% CI 0.97, 0.98], *p* < 0.001), a 2% decreased risk of cerebrovascular disease (OR 0.98 [95% CI 0.97, 0.99], *p* < 0.001) and a 3% decreased risk of peripheral vascular disease (OR 0.97 [95% CI 0.96, 0.99], *p* < 0.001) (Fig. 3). Each 1 year increase in age at diabetes diagnosis was associated with an 8% decreased risk of retinopathy (OR 0.92 [95% CI 0.90, 0.95], *p* < 0.001), a 6% decreased risk of nephropathy (OR 0.94 [0.92, 0.96], *p* < 0.001) and a 5% decreased risk of neuropathy (OR 0.95 [95% CI 0.94, 0.96], *p* < 0.001) (Fig. 3). Significant heterogeneity in the magnitude of the effects was evident between studies for these outcomes (all *χ*^2^ *p* < 0.001, all *I*^2^ ≥ 48%).

### Sensitivity analyses

#### Effects of age at diabetes diagnosis adjusted for diabetes duration on all-cause mortality, macrovascular disease and microvascular disease

Data for these analyses were obtained from ten studies comprising 390,139 participants and adjusted for diabetes duration (ESM Fig. 1). Each 1 year increase in age at diabetes diagnosis was associated with a 6% increased risk of all-cause mortality (OR 1.06 [95% CI 1.03, 1.09], *p* < 0.001), a 6% increased risk of macrovascular disease (OR 1.06 [95% CI 1.04, 1.07], *p* < 0.001) and a 5% increased risk of microvascular disease (OR 1.05 [95% CI 1.02, 1.08], *p* < 0.001).

### Methodological quality

Risk of bias assessment of the included studies is presented in ESM Table 3. Study participants were recruited to randomised clinical trials or selected from large clinical datasets. Inclusion and exclusion criteria were adequately described in all studies. Of the included studies, 24 [14, 15, 20, 21, 23–25, 36–52] were of high quality and two [53, 54] of medium quality due to insufficient adjustment of confounding variables. In addition, 24 studies [14, 15, 20, 21, 23–25, 36–52] demonstrated low risk of bias and two [53, 54] demonstrated a moderate risk of bias due to insufficient adjustment for confounding variables (confounding bias) (ESM Table 3). Funnel plots did not suggest the presence of publication bias (ESM Fig. 2).

## Discussion

This comprehensive systematic review and meta-analysis compiles the results of 26 studies investigating the effects of age at diabetes diagnosis on mortality and subsequent complications in 1,325,493 participants with type 2 diabetes from diverse populations across the Asia Pacific, Europe and North America. We report an inverse relationship between age at diabetes diagnosis and risk of major diabetes complications after adjustment for current age. Each 1 year increase in age at diabetes diagnosis was associated with a 4%, 3% and 5% decreased risk of all-cause mortality, macrovascular disease and microvascular disease, respectively. These effects were consistent across the individual components of the composite outcomes (CHD, cerebrovascular disease, peripheral vascular disease, retinopathy, nephropathy and neuropathy) and reversed when the models included diabetes duration rather than current age.

While earlier studies have assessed the effects of age at diabetes diagnosis on diabetes complications, to our knowledge, this is the first systematic review and meta-analysis exploring associations between age at diabetes diagnosis and subsequent outcomes. Interdependence of current age, age at diabetes diagnosis and diabetes duration precluded investigation of all three variables simultaneously; hence the use of models containing either age at diabetes diagnosis adjusted for current age or age at diabetes diagnosis adjusted for diabetes duration. People diagnosed with diabetes at an older age may be more likely to have accumulated adverse cardiovascular risk factors compared with those diagnosed at a younger age. Since advancing age is a powerful predictor of vascular complications, for the same diabetes duration, people diagnosed at a younger age are likely to have lower absolute risks of events as compared with people diagnosed at an older age. Over time, however, the effects of both ageing and disease duration may be amplified, resulting in premature complications and death in people diagnosed with type 2 diabetes at a younger age. To illustrate, a person diagnosed with type 2 diabetes at age 30 years would have a lower absolute risk of complications compared with a person diagnosed at age 50 years but by the time they both reach age 60 years the person diagnosed at a younger age would have a higher relative and absolute risk due to the effects of ageing, compounded by the effects of longer diabetes duration. This pattern has been observed in several young-onset type 2 diabetes populations [21, 55]. Thus, younger people pose a significant challenge for clinicians and decision makers who need to be aware of these compounding pathologies of natural ageing and premature vascular ageing associated with type 2 diabetes. Further, people diagnosed with type 2 diabetes at a younger age still have the potential to develop complications at an earlier stage of life, at a time when the complications are more likely to cause greater disability and loss of productivity compared with people diagnosed at an older age.

There is a lack of RCT studies on achieving good glycaemic control and optimisation of cardiovascular risk factors in young-onset type 2 diabetes; many trials recruited middle-aged people with long disease duration at greatest absolute risk of complications. However, data from these older populations may not reflect the pathophysiology of type 2 diabetes in younger people, given evidence suggesting that the development of diabetes complications may differ between younger and older individuals. Further, many of these studies lack sufficient follow-up to capture complications in younger people who may have a longer time to event. The observations of this study and others examining type 2 diabetes complications [15, 23, 38, 51] add impetus to conducting trials examining this young cohort. There is an urgent need for data specifically pertaining to younger type 2 diabetes populations examining the trajectory of vascular complications and the impact of interventions (pharmacological as well as non-pharmacological approaches) to improve outcomes.

We found that age at diabetes diagnosis adjusted for current age was inversely associated with risk of all-cause death, macrovascular disease and microvascular disease. Our findings underscore the importance of cardiovascular risk management among people with diabetes. Screening for and prevention of macrovascular complications is particularly important for older people with diabetes, who have the highest short-term absolute risk. Increasing age remains one of the most important risk factors for the development of macrovascular complications. However, it is also important to note that people diagnosed with diabetes at a younger age have a longer lifetime risk of developing significant complications, thus achieving good glycaemic control and optimisation of cardiovascular risk factors is of particular importance across their lifespan. This difference in risk between younger and older people in terms of absolute vs lifetime risks of type 2 diabetes complications, should perhaps be recognised in diabetes management guidelines, with increased promotion of screening programmes in older people with type 2 diabetes and a greater emphasis on preventive measures for younger people with type 2 diabetes.

As early intensive multifactorial risk factor intervention is important for the prevention of long-term macrovascular complications among people with newly diagnosed diabetes [56], our findings further suggest that this should be sustained long-term to minimise risks over time. Clearly, strategies are needed to ensure sustained adherence to lifestyle behaviours and therapies proven to have cardiovascular benefits among people with diabetes. Existing treatment guidelines are limited by being reactive to suboptimal glycaemic control after it has developed, without means to predict which people require intensified treatment. Refined stratification using age at diagnosis may provide a method of identifying, at diagnosis, those at greatest risk of complications who would most benefit from targeted, individualised treatment regimens. Moreover, public health measures to delay and/or prevent the onset of type 2 diabetes until older age may yield benefits by reducing the duration of diabetes and the burden of complications.

The development and progression of type 2 diabetes represents a complex interplay between genetic, epigenetic, lifestyle, demographic, socioeconomic, therapeutic and environmental factors. Given the myriad factors involved, and the variable reporting across included studies, it was difficult to establish uniformity in study definitions and covariate adjustment across studies. There was considerable variation in the definitions of ‘younger’ and ‘older’ age at type 2 diabetes diagnosis, with some studies defining ‘younger’ as >30 years of age, >40 years of age or >50 years of age. To mitigate this, we examined the effect of age at diabetes diagnosis (adjusted for current age), in yearly increments. Studies varied greatly with respect to measured confounding factors such as ethnicity, study country and year, type 2 diabetes diagnostic criteria, medication use, glycaemic control age, obesity, cardiometabolic risk factors, comorbid conditions, recruitment, source of participants, family history, healthcare access and sociodemographic factors. We were unable to adjust for these factors, as the data were either unavailable or not comparable due to the lack of standardised definitions across published studies. Moving forward, standardised approaches to reporting and complete data capture of relevant variables will assist with pooling and analysis of disparate datasets. This may be facilitated by the creation of international data registries. Performance bias (a potential difference in the care provided between early- and later-onset type 2 diabetes groups and between different centres) could not be assessed. Older people with type 2 diabetes may have cognitive impairment or other comorbidities precluding treatment intensification or even leading to de-intensification. Alternatively, people diagnosed with type 2 diabetes at a younger age may have been treated more intensively than people diagnosed at an older age. If this were the case, this bias would ameliorate the differences between groups, such that our data may actually underestimate the true extent of the effect of younger age at type 2 diabetes diagnosis. However, this appears less likely as several studies suggest that younger people with type 2 diabetes have poorer glycaemic control, lower adherence to therapy and inferior self-care practices compared with older people [57, 58]. In fact, the data suggest that younger people with type 2 diabetes receive suboptimal medical attention, potentially due in part to an absence of clinical guidelines targeted to younger people with type 2 diabetes and possibly the underestimation of risks of complications in these individuals [38].

The strength of this meta-analysis is the extensive and comprehensive literature search and focus on studies examining younger and older people with type 2 diabetes. Six databases were searched, a risk of bias appraisal performed, and reanalyses were undertaken, enabling inclusion of data from more than a million people with type 2 diabetes worldwide. Collaboration with other authors facilitated more homogeneous data definitions, data integration and meta-analysis. We found that there was high concordance between the different studies included in the meta-analysis, such that the directions of the effects were consistent, although the magnitude of effects and the CIs varied. This may be due to differences in study size, although contributions from genetic, ethnic and healthcare variations in study populations cannot be excluded. Nevertheless, the direction of the effects was consistent across the studies from different countries.

As with many systematic reviews and meta-analyses, this meta-analysis has some limitations. Not all identified studies were included in the meta-analysis due to difficulties sourcing comparable data from authors. The inability to acquire data from all eligible studies is not unexpected and is a part of the meta-analysis process [59]. We based our classification of age at type 2 diabetes diagnosis on the definitions used in each individual study, even though these definitions may have differed. It would be impossible to apply retrospectively a single definition of age at diagnosis to a large number of samples characterised with different variables in different studies. Additionally, the criteria for the diagnosis and classification of type 2 diabetes have changed with the advent of new technologies, such as the determination of pancreatic autoantibodies and C-peptide levels, as have the methods used to differentiate type 2 diabetes from other forms of diabetes (principally type 1 and monogenic diabetes). Lastly, due in part to the nature of the study question, the included studies were observational in design and therefore subject to the potential biases (confounding and selection) inherent to analyses of observational data. However, meta-analyses of observational studies can provide valuable insights, especially when RCTs are unavailable or are inappropriate for addressing the question [34], as is the case here. Findings from this review are based on observational data and therefore causality may not be attributed. Thus, although these findings may be applicable on a population level, any recommendations need to be individualised to the clinical situation of each person with type 2 diabetes.

We have completed the first systematic review and meta-analysis examining the effects of age at type 2 diabetes diagnosis on all-cause mortality, microvascular complications and macrovascular complications. This comprehensive analysis, comprising over a million participants, indicates that when adjusted for current age, younger age at type 2 diabetes diagnosis is associated with increased risk of mortality, macrovascular complications and macrovascular complications. Identification and quantification of the increased risk of mortality and vascular disease conferred by younger age at type 2 diabetes diagnosis may enable risk stratification of people early in the condition and thereby provide greater opportunities for interventions to reduce risk of complication-associated morbidity and mortality for this increasing population demographic developing type 2 diabetes.

## Supplementary Information

ESM 1 (PDF 238 kb)

## Data Availability

Application for datasets generated during and/or analysed during the current study may be considered by the corresponding author on reasonable request.
