# Supplementary material for: Impact of age at type 2 diabetes mellitus diagnosis on mortality and vascular complications: systematic review and meta-analyses
Source: Diabetologia. 2020 Dec 14;64(2):275–87. doi: 10.1007/s00125-020-05319-w (PMC7801294; doi:10.1007/s00125-020-05319-w)
Supplement: Supplementary file 1 — (PDF 238 kb) [file 125_2020_5319_MOESM1_ESM.pdf]

Electronic Supplementary Material: Impact of age at type 2 diabetes mellitus diagnosis on mortality and vascular complications: systematic review and meta-analyses

Methods - Search strategy: MeSH terms for Medline (equivalent searches used for other databases)

1 exp Diabetes Mellitus, Type 2/
2 (MODY or NIDDM or TIID or T2D*).mp.
3 (non insulin* depend* or noninsulin* depend* or noninsulin?depend* or non insulin?depend*).tw.
4 ((typ? 2 or typ? II or typ?2 or typ?II) adj3 diabet*).tw.
5 ((late or adult* or matur*) adj3 onset adj3 diabet*).tw.
6 or/1-5
7 exp Diabetes Insipidus/
8 diabet* insipidus.tw.
9 7 or 8
10 6 not 9
11 AGE OF ONSET.mp.
12 (age adj3 onset).mp.
13 AGE FACTORS.mp.
14 Age Factors/
15 (age adj3 diagnos*).mp.
16 or/11-15
17 (DISEASE adj3 PROGRESSION).mp.
18 Disease Progression/
19 Prognosis/
20 PROGNOSIS.mp.
21 or/17-20
22 10 and 16 and 21
23 exp Diabetes Complications/
24 exp Diabetes Mellitus, Type 2/co
25 23 or 24
26 10 and 16 and 25
27 22 or 26
28 limit 27 to english language
29 exp animals/ not humans.sh.
30 28 not 29
31 30 or 22

* The numbers in the search strategy correspond to the line number of the query

ESM Table 1

PICO – Inclusion/ Exclusion criteria

	Participants (P)	Intervention (I)	Comparison (C)	Outcomes (O)	Study Type	Limits
Inclusion Criteria	Adults with Type 2 Diabetes (>18 years)	Nil, Observational Studies	Early Vs Late Onset Diabetes<br>Comparable Duration<br>Subgroups	All-cause mortality<br>Retinopathy<br>Nephropathy<br>Neuropathy<br>Cardiovascular Disease<br>Cerebrovascular Disease<br>Peripheral Vascular Disease<br>Cognitive Impairment<br>Frailty	Observational<br>Case Control<br>Prospective<br>Comparator Group	English articles<br><br>No time restriction<br><br>Human subjects
Exclusion Criteria	No Diabetes<br>Type 1 Diabetes<br>Other Diabetes Eg Type 1, Gestational, Secondary, Monogenic<br>Diabetes Insipitus	Nil	Other Factors eg Glycaemic Control	Outcomes Unrelated To Diabetes Complications	Editorials<br>Letters<br>Narrative Reviews	Non-English Articles<br>Non-Human Subjects

ESM Table 2 – Risk of bias

Table 2: Risk of bias ratings (Centre for Clinical Effectiveness, 2010).

Rating	Description
**Low (L)**	All of the criteria have been fulfilled or where criteria have not been fulfilled it is very unlikely the conclusions of the study would be affected
**Moderate (M)**	Some of the criteria have been fulfilled and those criteria that have not been fulfilled may affect the conclusions of the study
**High (H)**	Few or no criteria fulfilled or the conclusions of the study are likely or very likely to be affected

ESM Table 3: Risk of bias assessment

Study Authors	*Selection bias, study participants representative*	*Detection bias*		*Nil reporting bias, free of selective outcome reporting*	*Assessment of confounding in original analysis*	*Conflict of interest*	*Overall risk of bias*
		Adequate exposure measures	*Adequate outcome measures*				
Amutha A, Anjana RM., et al	Yes	Yes	Yes	Yes	Yes	No	Low
Amutha A, Datta M, et al	Yes	Yes	Yes	Yes	Yes	No	Low
Cai, X., et al.	Yes	Partial	Yes	Yes	Yes	No	Low
Chan, J.C., et al	Yes	Yes	Yes	Yes	Yes	No	Low
Chen, M.S., et al	Partial	Partial	Yes	Yes	Yes	NR	Medium
Hamman, R.F., et al	Yes	Partial	Yes	Yes	Yes	NR	Medium
Huo, L., et al	Yes	Yes	Yes	Yes	Yes	No	Low
Huo, X., et al	Yes	Yes	Yes	Yes	Yes	No	Low
Kenealy, T., et al	Yes	Yes	Yes	Yes	Yes	No	Low
Nanayakkara, N., et al	Yes	Yes	Yes	Yes	Yes	No	Low
Pavkov, M.E., et al	Yes	Yes	Yes	Yes	Yes	No	Low
Pradeepa R, Anjana RM., et al	Yes	Yes	Yes	Yes	Yes	No	Low
Pradeepa R, Chella S., et al	Yes	Yes	Yes	Yes	Yes	No	Low
Pradeepa R, Rema M., et al	Yes	Yes	Yes	Yes	Yes	No	Low
Pugliese, G., et al	Yes	Yes	Yes	Yes	Yes	No	Low
Pugliese, G., et al	Yes	Yes	Yes	Yes	Yes	No	Low
Rema, M., et al	Yes	Yes	Yes	Yes	Yes	No	Low
Romero-Aroca, P., et al	Yes	Yes	Yes	Yes	Yes	No	Low
Song, S.H. and C.A. Hardisty	Yes	Yes	Yes	Yes	Yes	No	Low
Song, S.H. and T.A. Gray	Yes	Yes	Yes	Yes	Yes	No	Low
Thomas, R.L et al	Yes	Yes	Yes	Yes	Yes	No	Low
Unnikrishnan, R. Anjana., et al	Yes	Yes	Yes	Yes	Yes	No	Low
Unnikrishnan, RI, Rema M., et al	Yes	Yes	Yes	Yes	Yes	No	Low
Wong, J., et al	Yes	Yes	Yes	Yes	Yes	NR	Low
Yeung, R.O.,	Yes	Yes	Yes	Yes	Yes	No	Low
Zoungas, S., et al	Yes	Yes	Yes	Yes	Yes	No	Low

NR = not reported

ESM Figure 1 - Age at diabetes diagnosis adjusted for diabetes duration

Primary Outcomes

All-cause mortality

Study or Subgroup	log[Odds Ratio]	SE	Total	Total	Weight	Odds Ratio IV, Random, 95% CI
Kenealy, T., et al	0.076035	0.002602	0	70057	25.3%	1.08 [1.07, 1.08]
Pavkov, M.E., et al	0.04953	0.002868	0	2963	25.2%	1.05 [1.04, 1.06]
Penno, G., et al	0.027615	0.001489	0	15733	25.4%	1.03 [1.03, 1.03]
Zoungas, S., et al	0.09531	0.00699	0	11140	24.0%	1.10 [1.09, 1.12]
**Total (95% CI)**			**0**	**99893**	**100.0%**	**1.06 [1.03, 1.09]**

Heterogeneity: Tau² = 0.00; Chi² = 329.08, df = 3 (P < 0.00001); I² = 99%
Test for overall effect: Z = 4.36 (P < 0.0001)

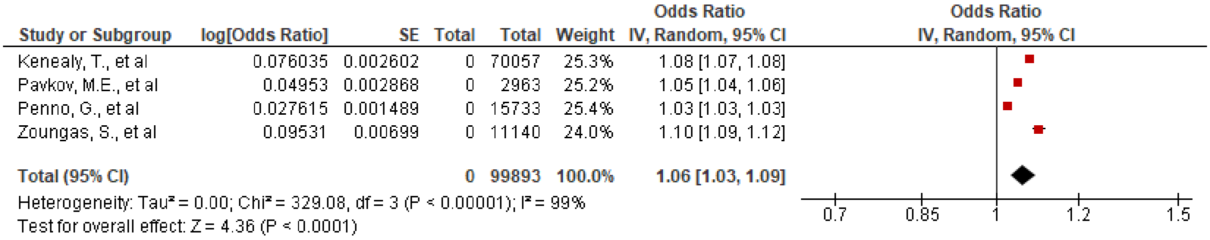

Macrovascular disease

Study or Subgroup	log[Odds Ratio]	SE	Total	Total	Weight	Odds Ratio IV, Random, 95% CI
Chan, J.C., et al	0.067863	0.004925	0	9506	11.3%	1.07 [1.06, 1.08]
Chuang, L.M., et al	0.067659	0.016617	0	8054	7.2%	1.07 [1.04, 1.11]
Huo, X., et al	0.04879	0.000729	0	222770	11.9%	1.05 [1.05, 1.05]
Kenealy, T., et al	0.039221	0.001716	0	48444	11.8%	1.04 [1.04, 1.04]
Nanayakkara, N., et al	0.037819	0.003854	0	2995	11.5%	1.04 [1.03, 1.05]
Pugliese, G., et al	0.028587	0.001983	0	15933	11.8%	1.03 [1.03, 1.03]
Song, S.H. and C.A. Hardisty	0.04879	0.004859	0	2733	11.3%	1.05 [1.04, 1.06]
Yeung, R.O.,	0.094001	0.001951	0	42453	11.8%	1.10 [1.09, 1.10]
Zoungas, S., et al	0.058269	0.004813	0	11140	11.3%	1.06 [1.05, 1.07]
**Total (95% CI)**			**0**	**364028**	**100.0%**	**1.06 [1.04, 1.07]**

Heterogeneity: Tau² = 0.00; Chi² = 694.67, df = 8 (P < 0.00001); I² = 99%
Test for overall effect: Z = 7.63 (P < 0.00001)

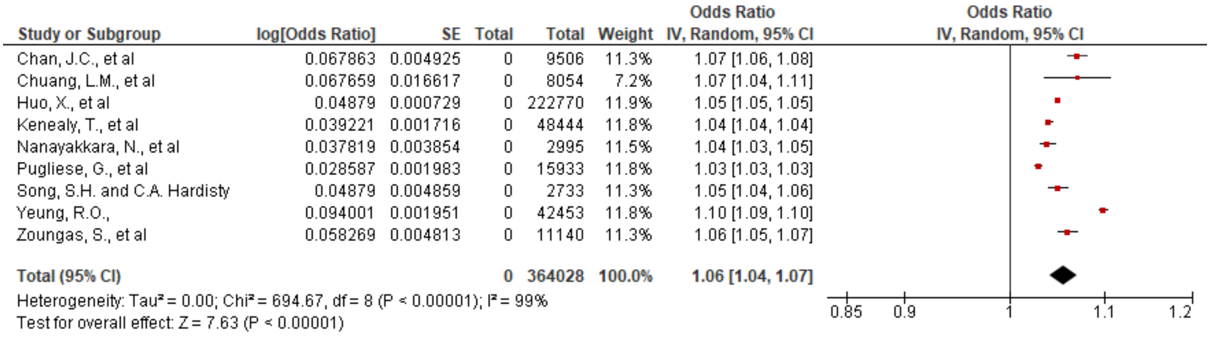

Microvascular disease

Study or Subgroup	log[Odds Ratio]	SE	Total	Total	Weight	Odds Ratio IV, Random, 95% CI
Amutha A, Anjana RM., et al	0.122218	0.065538	0	90	3.3%	1.13 [0.99, 1.28]
Chan, J.C., et al	0.137685	0.003994	0	9506	12.2%	1.15 [1.14, 1.16]
Huo, X., et al	0.0139	0.000755	0	222770	12.3%	1.01 [1.01, 1.02]
Kenealy, T., et al	0.039221	0.001716	0	54157	12.3%	1.04 [1.04, 1.04]
Nanayakkara, N., et al	0.007818	0.003485	0	3033	12.2%	1.01 [1.00, 1.01]
Pradeepa R, Anjana RM., et al	0.058269	0.014444	0	1608	10.9%	1.06 [1.03, 1.09]
Pugliese, G., et al	0.026642	0.00174	0	15733	12.3%	1.03 [1.02, 1.03]
Yeung, R.O.,	0.094818	0.001637	0	42453	12.3%	1.10 [1.10, 1.10]
Zoungas, S., et al	-0.01005	0.005154	0	11140	12.1%	0.99 [0.98, 1.00]
**Total (95% CI)**			**0**	**360490**	**100.0%**	**1.05 [1.02, 1.08]**

Heterogeneity: Tau² = 0.00; Chi² = 2887.99, df = 8 (P < 0.00001); I² = 100%
Test for overall effect: Z = 3.47 (P = 0.0005)

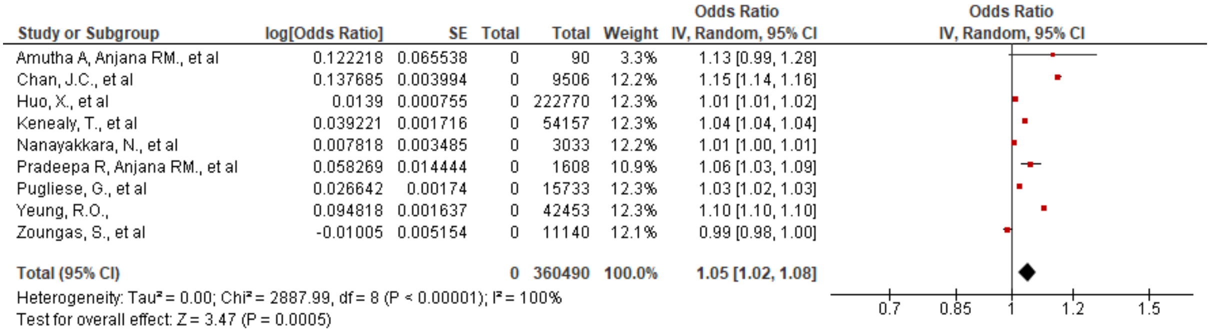

ESM Figure 1: Effect of age at diagnosis (as a continuous variable), adjusted for diabetes duration on the risk of all-cause mortality, macrovascular and microvascular disease. The symbols are proportional to the study weight and horizontal lines represent 95% confidence intervals.

ESM Figure 2 - Publication bias

All-cause mortality

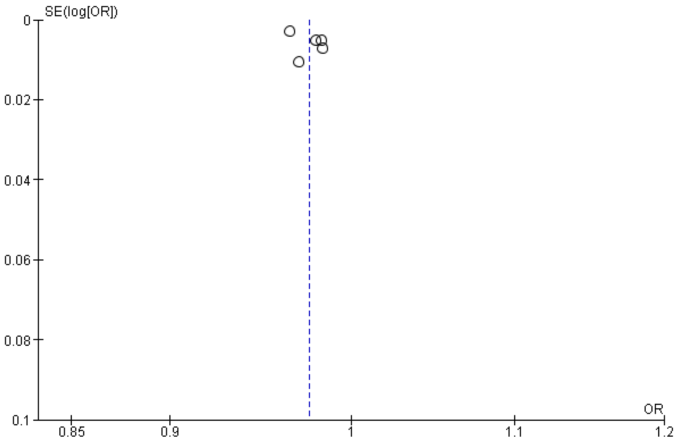

Macrovascular disease

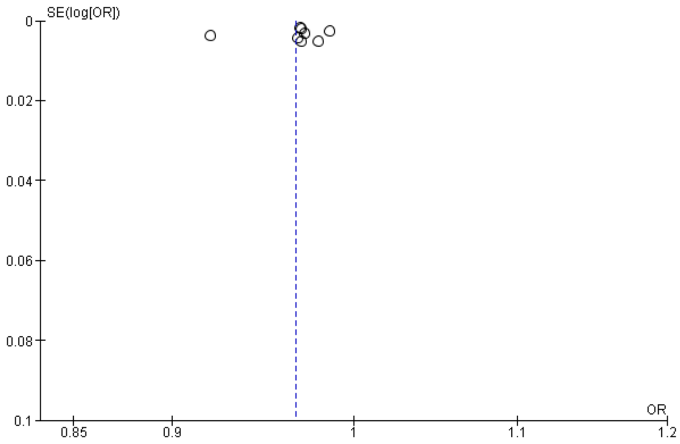

Microvascular disease

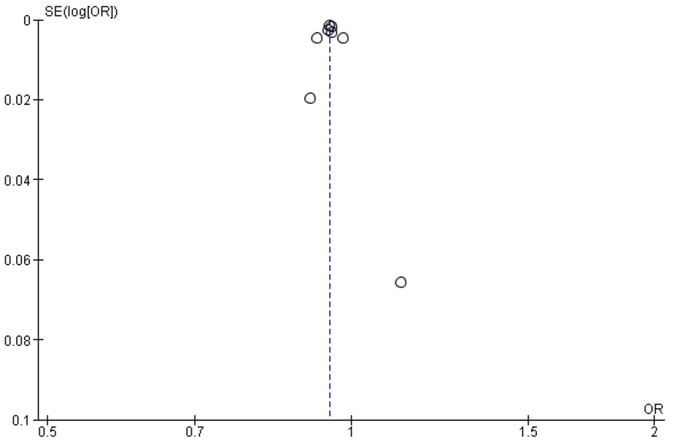

ESM Figure 2: Funnel plot of comparison, all-cause mortality, macrovascular and microvascular disease. Dotted line represents effect estimate. Circles represent individual studies.
